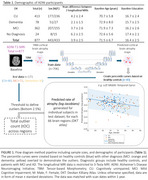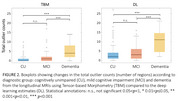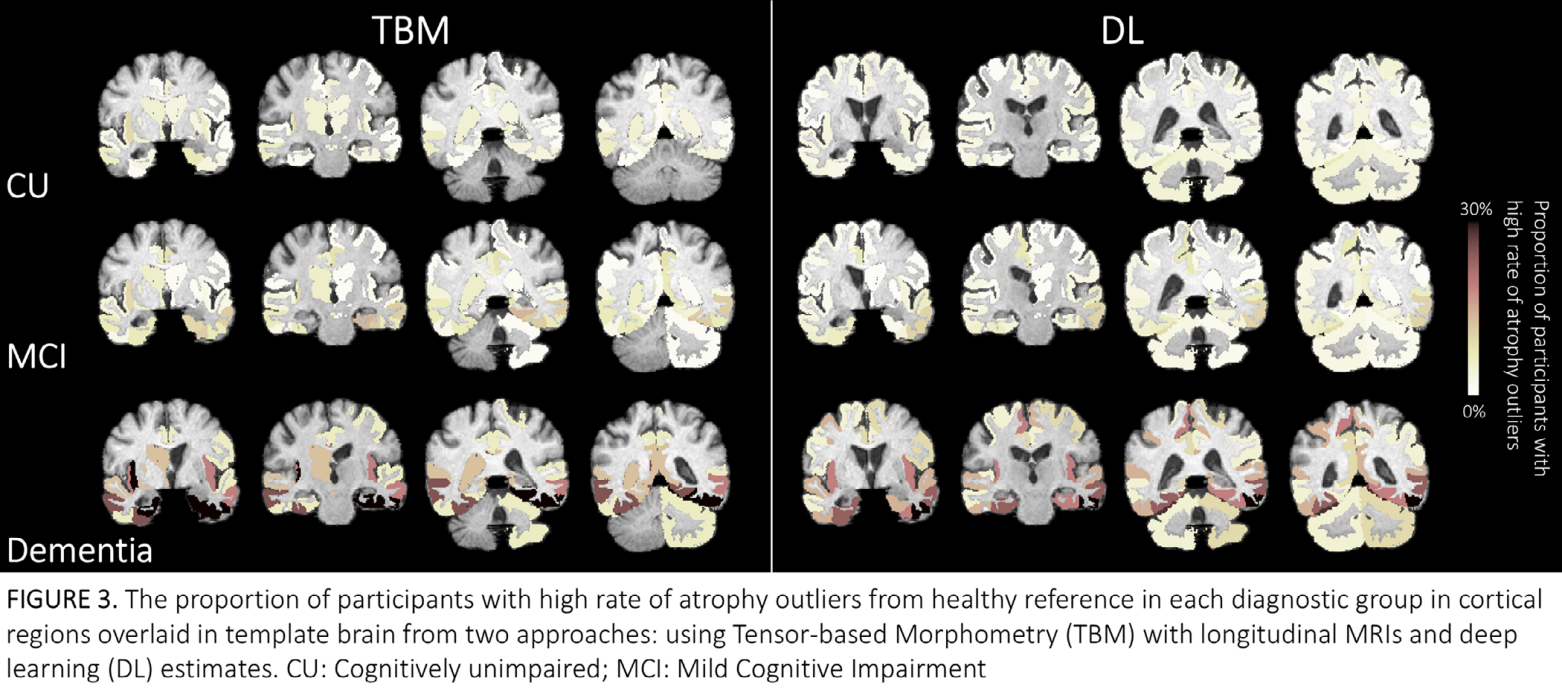# Identifying abnormal brain atrophy in Alzheimer’s Disease using deep learning‐based MRI

**DOI:** 10.1002/alz70861_108230

**Published:** 2025-12-23

**Authors:** Linh N. N. Le, Evan Fletcher, Duygu Tosun, Jinyi Qi, Audrey P. Fan

**Affiliations:** ^1^ Department of Biomedical Engineering, University of California, Davis, Davis, CA USA; ^2^ Department of Neurology, University of California, Davis, Davis, CA USA; ^3^ Department of Radiology and Biomedical Imaging, University of California, San Francisco, San Francisco, CA USA; ^4^ Department of Electrical and Computer Engineering, University of California, Davis, Davis, CA USA

## Abstract

**Background:**

Given the heterogenous and nonlinear nature of Alzheimer’s Disease progression, characterizing individual neurodegeneration trajectories is crucial for identifying participants at the right disease stage for clinical trials. We developed a deep learning‐based (DL) model to predict two‐year longitudinal cortical atrophy rates from baseline MRI and aimed to characterize abnormal, high atrophy rate outlier patterns across the Alzheimer’s Disease Neuroimaging Initiative (ADNI). We hypothesized that DL derived longitudinal atrophy rate patterns could capture known patterns of atrophy associated with cognitive impairment syndromes.

**Method:**

We used two‐serial 3T‐T1‐weighted MRI scans (≥1 year apart) from ADNI participants (training: n=706; testing: n=83 CU, n=72 MCI, n=16 dementia; demographics in Table 1). Images were preprocessed, co‐registered to MDT template, and parcellated into 66 cortical regions using the Desikan‐Killiany atlas. A UNET architecture was trained on baseline MRIs to predict 2‐year atrophy rate maps (log‐Jacobians from Tensor‐based Morphometry [TBM]). Using predicted atrophy maps from controls, we established reference percentile curves for each brain region (covarying for age and sex). For each participant in the test cohort (CU, MCI, dementia), we identified brain regions with high rates of atrophy outliers, defined as predicted atrophy rates lower than 2.5^th^ percentile of the healthy reference. The total outlier count (tOC) was the sum of participant’s outlier regions. We then compared the estimates from the longitudinal MRIs using TBM as the ground truth to DL estimates.

**Result:**

tOC estimated from DL model significantly differed between CU (2.2±4.0), MCI (2.9±3.6), and dementia (8.4±5.7) groups, with significant differences found between CU and dementia (*p* <0.0001) and MCI and dementia (*p* <0.0001), consistently with the trend using TBM (Figure 2). In both models, dementia group showed a greater number of rates of atrophy outliers, compared to other groups. Regions where over 30% of dementia participants showed high atrophy rate outliers were predominantly located within the medial temporal cortex, including parahippocampal, entorhinal, and middle temporal areas (Figure 3).

**Conclusion:**

Our deep learning model successfully identified individuals with abnormal, high rates of cortical atrophy from baseline imaging. This approach provides a sensitive biomarker to capture individual differences in disease progression, potentially aiding in subject selection and monitoring in clinical trials.